# Red to Brown: An Elevated Anthocyanic Response in Apple Drives Ethylene to Advance Maturity and Fruit Flesh Browning

**DOI:** 10.3389/fpls.2019.01248

**Published:** 2019-10-09

**Authors:** Richard V. Espley, Davin Leif, Blue Plunkett, Tony McGhie, Rebecca Henry-Kirk, Miriam Hall, Jason W. Johnston, Matthew P. Punter, Helen Boldingh, Simona Nardozza, Richard K. Volz, Samuel O’Donnell, Andrew C. Allan

**Affiliations:** ^1^Plant & Food Research, Auckland, New Zealand; ^2^Plant & Food Research, Palmerston North, New Zealand; ^3^Hawke’s Bay Research Centre, Plant & Food Research, Havelock North, New Zealand; ^4^Plant & Food Research, Hamilton, New Zealand; ^5^School of Biological Sciences, University of Auckland, Auckland, New Zealand

**Keywords:** apple, anthocyanin, flavonoids, ethylene, peroxidase, transcription factors, enzymatic browning, ripening

## Abstract

The elevation of anthocyanin contents in fruits and vegetables is a breeding target for many crops. In some fruit, such as tomato, higher anthocyanin concentrations enhance storage and shelf life. In contrast, highly anthocyanic red-fleshed apples (*Malus* x *domestica*) have an increased incidence of internal browning flesh disorder (IBFD). To determine the mechanisms underlying this, ‘Royal Gala’ cultivar apples over-expressing the anthocyanin-related transcription factor (TF) MYB10 (35S:MYB10), which produces fruit with highly pigmented flesh, were compared with standard ‘Royal Gala’ Wild Type (WT) grown under the same conditions. We saw no incidence of IBFD in WT ‘Royal Gala’ but the over-expression of MYB10 in the same genetic background resulted in a high rate of IBDF. We assessed concentrations of potential substrates for IBDF and a comparison of metabolites in these apples showed that anthocyanins, chlorogenic acid, pro-cyanidins, flavon-3-ols, and quercetin were all higher in the MYB10 lines. For the flavol-3-ols sub-group, epicatechin rather than catechin was elevated in MYB10 lines compared with the control fruit. Internal ethylene concentrations were measured throughout fruit development and were significantly higher in 35S:MYB10 lines, and ethylene was detected at an earlier developmental stage pre-harvest. Expression analysis of key genes associated with ethylene biosynthesis (aminocyclopropane-1-carboxylic acid synthase and oxidase; *ACS* and *ACO*) and polyphenol oxidase (*PPO*) showed the potential for increased ethylene production and the mechanism for enhanced PPO-mediated browning. The expression of a transcription factor of the ethylene response factor (ERF) class, *ERF106*, was elevated in red flesh. Analysis of transcriptional activation by MYB10 showed that this transcription factor could activate the expression of apple *ACS*, *ACO*, and *ERF106* genes. Our data show a link between the elevation of anthocyanin-related transcription factors and an undesirable fruit disorder. The accelerated advancement of maturity *via* premature ethylene induction has implications for the breeding and storage of these more highly pigmented plant products.

## Introduction

Apples have a long history of association with human civilisation and over 8,000 years of domestication. The current domesticated apple (*Malus* x *domestica*) has become a fruit with high economic value with over 83 million tonnes harvested worldwide (FAO, 2018). Novel cultivars continue to be bred, such as red-fleshed apples created by crossing wild-red fleshed apples with domesticated varieties (Espley et al., 2007; Volz et al., 2009). While large red-fleshed apples are now available, they suffer from a variety of fruit quality issues. Most notably, red-fleshed apples suffer from an increased incidence in enzymatic browning (Volz et al., 2013).

In fruit development, ripening represents the final stage. During this period, important structural, biochemical, and physiological changes such as net starch degradation, softening of the flesh, changes in aroma and flavour profiles occur (Giovannoni et al., 2017). Additionally, two types of fruit ripening behaviour are present amongst plants, climacteric and non-climacteric (Lelievre et al., 1997). Climacteric fruits usually undergo a large burst of ethylene just before, during, or after a respiratory peak also known as a climacteric rise (Wills et al., 2001). Apples fall under this category. Non-climacteric fruits do not exhibit the large burst of ethylene nor any changes in respiration. Fruits that fall under this category include citrus (Alonso et al., 1995), grape, and strawberry (Giovannoni, 2004).

Apple fruit, such as ‘Royal Gala’, reach the stage of commercial harvest maturity at around 130 days after full bloom (Janssen et al., 2008), although maturity ranges from around 100 to 190 days after full bloom (DAFB), depending on cultivar and climate. In this phase, cell wall modifying enzymes are produced, causing changes in texture which makes the fruit palatable (Schaffer et al., 2013). This causes cell wall polysaccharides, including pectin and cellulose, to be broken down by cell wall degrading enzymes such as polygalacturonase. Changes in flavour are mainly due to a change in sugar-acid balance, a breakdown of bitter compounds (tannins and flavonoids) and an increase in production of volatiles such as methyl esters (ocimene and myrcene) to a total of at least 34 esters upon ripening in ‘Royal Gala’ (Young et al., 2004; Defilippi et al., 2009). Apple also undergoes a colour change as a result of the reduced production of chlorophyll and its degradation causing the unmasking of pigments that were already previously formed and synthesis of new pigments (carotenoids or anthocyanins) (Ferrer et al., 2005).

Browning (enzymatic browning) is a reaction that often occurs in fruit at the end of ripening and the beginning of over-ripening (Murata et al., 1995). Apple fruit, because of their high phenolic content, are highly susceptible to browning (Holderbaum et al., 2010). Internal browning can be triggered when the fruit is wounded (Queiroz et al., 2008). Browning is also related to abiotic stress in storage, such as low temperature, low oxygen, and/or high carbon dioxide (Mellidou et al., 2014). Browning occurs when phenolic compounds are oxidised by the enzyme polyphenol oxidase (PPO) which causes brown pigments to be generated (Queiroz et al., 2008). PPOs belong to a large gene family and of these, ten PPO genes have been mapped to the ‘Golden Delicious’ apple genome (Di Guardo et al., 2013). Polyphenol compounds are stored in vacuoles whilst PPOs are found in plastids (Holderbaum et al., 2010). Wounding, such as cutting or dropping the fruit, can weaken the cells, which allows both PPO and phenolic compounds to interact, causing polyphenols to revert to their corresponding quinones when oxidised by PPO. These then polymerise with other quinones or phenols to form brown pigments (Murata et al., 1995). Similarly, ripening also causes weakening effects in cells because enzymes, such as polygalacturonases, break down cell walls (Kramer and Redenbaugh, 1994). In apple cultivars, such as ‘Aori27’ and ‘Mellow’, chlorogenic acid is the major phenolic compound that is oxidised. In ‘Fuji’, chlorogenic acid and epicatechin are the major phenolic compounds, whilst in ‘Elstar’, epicatechin and procyanidin predominate (Holderbaum et al., 2010). The activity of PPO has also been shown to decrease as the apple ripens. This is due to a denaturing of the protein and not reduced PPO production (Murata et al., 1995). The ability to control enzymatic browning is important because it negatively impacts on colour, taste, flavour, and nutritional value in many fruits and vegetables (Holderbaum et al., 2010). Recently one apple has been engineered to reduce enzymatic browning. Named the ‘Artic’ apple, it possesses a non-browning trait conferred to it by silencing the PPO through RNA interference (RNAi), therefore reducing its expression (Waltz, 2015). This results in an apple which retains its colour, taste, flavour, and nutritional value even when damaged or cut.

Anthocyanins are a class of flavonoids commonly found in fruits and vegetables responsible for the vivid red, blue, and purple colours commonly found in nature (He and Giusti, 2010). In apple, the anthocyanins are primarily composed of cyanidin-3-galactoside with cyanidin-3-arabinoside, cyanidin-7-arabinoside, and cyanidin-3-xyloside usually in minor amounts (Vrhovsek et al., 2004; Meng et al., 2016). Overall, anthocyanins compose around 1–3% of total polyphenols in apples (Vrhovsek et al., 2004).

Anthocyanins are generated by enzymes of the phenylpropanoid pathway, including chalcone synthase (CHS) and chalcone isomerase (CHI), flavanone 3-hydroxylase (F3H), flavonoid 3’-hydroxylase (F3’H), flavonoid 3’,5’-hydroxylase (F3’5’H) dihydroflavonol reductase (DFR), anthocyanidin synthase (ANS), and leucoanthocyanidin dioxygenase (LDOX) flavonoid-3-O-glycosyltransferase (UFGT/UF3GT) (Holton and Cornish, 1995; Winkel-Shirley, 2001; Tanaka et al., 2008). These enzymes are regulated at the transcriptional level by a well-studied complex termed the MBW complex (proteins of the transcription factor [TF] classes MYB, basic helix-loop-helix bHLH and a WD40 repeat protein). In apple, MYB10, which is allelic to MYB1/MYBA, regulates anthocyanin biosynthesis, having 58% overall protein identity to Production of Anthocyanin Pigment 1 (PAP1/MYB75) in Arabidopsis. The apple bHLHs MdbHLH3 and MdbHLH33 are both members of the IIIf subfamily (Heim et al., 2003) and interact with MdMYB10. Both share high homology with TT8 in Arabidopsis and Delila in snapdragon (Espley et al., 2007).

In addition to the MBW complex, other TFs affect anthocyanin biosynthesis *via* protein-protein interaction with the MBW complex, or regulation of expression levels of genes encoding the MBW. A SQUAMOSA MADS-box gene regulates anthocyanin accumulation in bilberry (Jaakola et al., 2010). More recently, ethylene response factors (ERFs) have been shown to affect fruit colour at this level. In apple, MdERF1B has been shown to bind to the promoters of anthocyanin and proanthocyanidin regulating MYBs to alter anthocyanin and proanthocyanidin concentration (Zhang et al., 2018). In pear, PyERF3 interacts with PyMYB114 and its partner PybHLH3 to co-regulate anthocyanin biosynthesis (Yao et al., 2017) while in apple MYB1/10 was shown to activate expression of apple ERF3 (a close homologue to pear ERF3) and this increase in ERF expression increases ethylene emission from apple callus (An et al., 2018). Furthermore, it was shown that EIL1 directly bound to the promoter of MYB1 to induce anthocyanin production, while MYB1 was able to interact with ERF3, providing a positive feedback loop for ethylene biosynthesis. The apple bHLH3, a known partner to MYB1/10 in the MBW complex, has also been implicated in activating ACO and ACS1 and ACS5 genes to drive ethylene production (Hu et al., 2019). In a recent study of the transcriptome of red-fleshed apple (Wang et al., 2018), WRKY11 and ERF106 were identified as being differentially regulated and both were more expressed in red flesh.

Here, we use isogenic lines of ‘Royal Gala’ that differ genetically by only the over-expression of *MYB10* (Espley et al., 2007). These lines show that MYB10 can activate the expression of apple *ACS* and *ACO* genes, possibly *via* up-regulation of *ERF* genes. This activation links anthocyanin concentrations and ethylene, as well as enhanced amounts of PPO and substrates for the browning reaction. This linkage has implications for the quality of highly pigmented apples.

## Materials and Methods

### Plant Materials

To generate highly anthocyanic apple fruit, the *Malus* x *domestica* ‘Royal Gala’ apple cultivar was transformed with an over-expression construct containing *MYB10* cDNA under the control of the CaMV35S promoter as previously described (Espley et al., 2007). Fruit from multiple trees for three independent transgenic lines (A1, A3, and A4) over two seasons (2013–2014 and 2014–2015) were used in this study and compared with non-transformed wild type (WT) ‘Royal Gala’ fruit grown under the same conditions in a containment glasshouse. Samples from each season were collected at seven different time points: T1 = 35 Days After Full Bloom (DAFB); T2 = 65DAFB; T3 = 85 DAFB; T4 = 110 DAFB = T5, 120 DAFB; T6 = 130 DAFB (WT ‘Royal Gala’ commercial maturity); T7, 140 DAFB, using fruit from two or three transgenic lines (A1, A3, A4; dependent on fruit number) and WT control fruit. For gene expression analysis T2 to T6 were assayed, to capture the most relevant developmental stages.

### Fruit Maturity and Ripening Assessments

Ethylene concentrations were determined both during fruit development and at harvest on three transgenic lines (A1, A3, and A4) and ‘Royal Gala’ WT fruit. For fruit *on planta*, a needle was inserted into the core cavity and the insertion site was sealed with wax to prevent wounding-related damage or gaseous loss; this remained in place during fruit development. Internal ethylene concentration was determined by extracting a 1 ml core cavity gas sample, using a compatible syringe to the inserted needle, and injecting it into a gas chromatograph (Hewlett Packard, 5890 series II) as previously described (Johnston et al., 2009). For harvested fruit, ethylene samples were extracted at one of the developmental time points using the same analysis method with five biological replicates. Fruit firmness was assessed by Texture Analyser TAXT plus (Stable Microsystems, United Kingdom) fitted with a 7.9-mm Effegi penetrometer probe (Johnston et al., 2009). Soluble solids content (SSC) was assessed by hand-held refractometer. Fruit were also maintained in industry-standard storage conditions for ten weeks after harvest at 0.5°C and assessed for extent of internal browning.

### Peroxidase Enzyme Assay

Enzyme activity was assessed spectrophotometrically at 25°C. All assays were performed on a SpectroMax plus 384 UV-vis spectrophotometer and analysed using the SoftMax Pro v5.4.5 software. Peroxidase (POX) was extracted and assayed based on a modified version previously described (Lester et al., 2004). Apple cortical tissue was frozen and ground into a fine powder in liquid nitrogen; 250 mg of tissue was then homogenised in 1 ml of 100 mM phosphate buffer pH 7.0 containing 0.5 mM cysteine and 4% w/v polyvinylpolypyrrolidone (PVPP). POX activity was determined by adding 25 µL of the extract to 225µL of assay reagent (50 mM potassium phosphate, pH 7.0, 10 mM guaiacol, and 10 mM H_2_O_2_) in a 96-well plate. The reaction was initiated by adding 10 mM guaiacol, and the activity determined by the rate of formation of tetraguaiacol at 470 nM. Assays were performed in triplicate for each of the biological replicates. Results are presented as the maximum rate of change per mg of protein.

### Non-Structural Carbohydrates and Anthocyanin Analysis

Fruit flesh tissue was ground under liquid nitrogen using an IKA (IKA, Staufen, Germany) grinder and stored at -80ºC. Soluble sugars were extracted from 200 mg aliquots of ground flesh in 80% ethanol. The soluble sugars (glucose, fructose, sucrose, and sorbitol) were analysed by ion chromatography as described in Nardozza et al. (2013). Starch was analysed by a colorimetric method following enzymatic digestion of the pellet obtained from the sugar extraction, as previously described by Smith et al. (1992). Anthocyanin and flavonoids were extracted in methanol (75% in water) and quantified by High Performance Liquid Chromatography (HPLC), using a method previously described (Espley et al., 2013). Starch accumulation rate/degradation was calculated using the Relative Growth Rate (RGR) where the primary data were logarithmically transformed to homogenise variability (Opara, 2000).

### Gene Expression Analysis

RNA from apple flesh was isolated using the Spectrum^™^ Plant Total RNA kit (Sigma) and cDNA was synthesised using the Qantitect^®^ Reverse Transcription kit (Qiagen), both according to the manufacturer’s recommendations. Primers used for qPCR analysis was designed using Geneious 8.1.9 (Kearse et al., 2012) and synthesised by Macrogen (Republic of Korea). Primers are listed in **Table S1**. qPCR analysis was performed using the LightCycler^®^ 480 using LightCycler FastStart SYBR Green Mix (Roche Diagnostics), according to the manufacturer’s guideline. Reactions contained 2.5 μl Master Mix, 0.25 μl of each primer (10 mM), 1.25 ml diluted cDNA (1:25), and nuclease-free water (Roche Diagnostics) to a total volume of 5 μl, using reaction conditions previously reported (Espley et al., 2007). Expression was normalised against *Malus* x *domestica Elongation factor 1* (*MdEF1α*), a ‘housekeeping’ gene known for its consistent transcript level in apple fruits and leaves (Chagné et al., 2013).

### Isolation and Cloning of Apple Gene Promoter Sequences

For the cloning of apple promoters used in the Dual Luciferase assays, nested primers were designed to the target sequence. The inner primers for use in promoter isolation were designed to isolate approximately 2 kb upstream of the transcription start site of each gene. PCR fragments were cloned using Platinum^®^ Taq DNA Polymerase High Fidelity (Thermofisher Scientific) as per manufacturer’s protocols. For all candidate gene promoters, outer PCR amplification was first performed and checked on agarose gels. The PCR product was diluted 100 x and 1.5 μl of this was used as the template for the inner PCR amplification. Fragments were cleaned using DNA Clean and Concentrator™ (Zymo Research) as per manufacturer’s protocol and cloned into the pGreenII 0800-Luc using the In-Fusion^®^ HD Cloning Kit. Promoter fragments were verified by sequencing. Analysis of putative binding TF sites in the MdERF106 promoter was carried out using PlantPAN 2.0 (Chien et al., 2015). The MYB TF over-expression constructs used in the luciferase assays were as previously described (Lin-Wang et al., 2011; Brendolise et al., 2017).

### Dual Luciferase Assays

Glasshouse-grown *Nicotiana benthamiana* plants were used for the dual luciferase assays to determine transcription factor activation of promoter sequences, as previously described (Hellens et al., 2005).

### Phylogenic and Statistical Analysis

Arabidopsis and other plant protein sequences were obtained from NCBI. Full-length sequences were aligned using MEGA 6.06 (Tamura et al., 2013) MUSCLE (open = -2.9, gap = 0) (Edgar, 2004). Phylogenetic analysis of the proteins were performed using MEGA 6.06 using a maximum likelihood method based on the JTT matrix-based model (Jones et al., 1992) and 1,000 bootstrap replicates. Analysis of the ERF genes was performed using MEGA 6.06 (6140226) *via* the Neighbour-Joining method (Saitou and Nei, 1987) with evolutionary distances calculated using the JTT matrix-based model and 1,000 bootstrap replicates.

The effects of the transgenic modification and fruit developmental stage (factors) on secondary metabolites, ethylene, peroxidase enzyme activity, and non-structural carbohydrates were analysed by ANOVA (type 3 sums of squares Kenward-Roger’s method) using a linear mixed effects model in R (version 3.5.1). The effect of transcription factors (which is the factor in the analysis) on activating a selected promoter was analysed with the same method. The biological replicates were treated as random effects. The means were separated on the base of all pairwise comparisons of least-squares means (letters assigned).

## Results

### Elevated Anthocyanin Content Causes a Higher Incidence of Internal Browning Flesh Disorder (IBFD)

**Figure 1:**
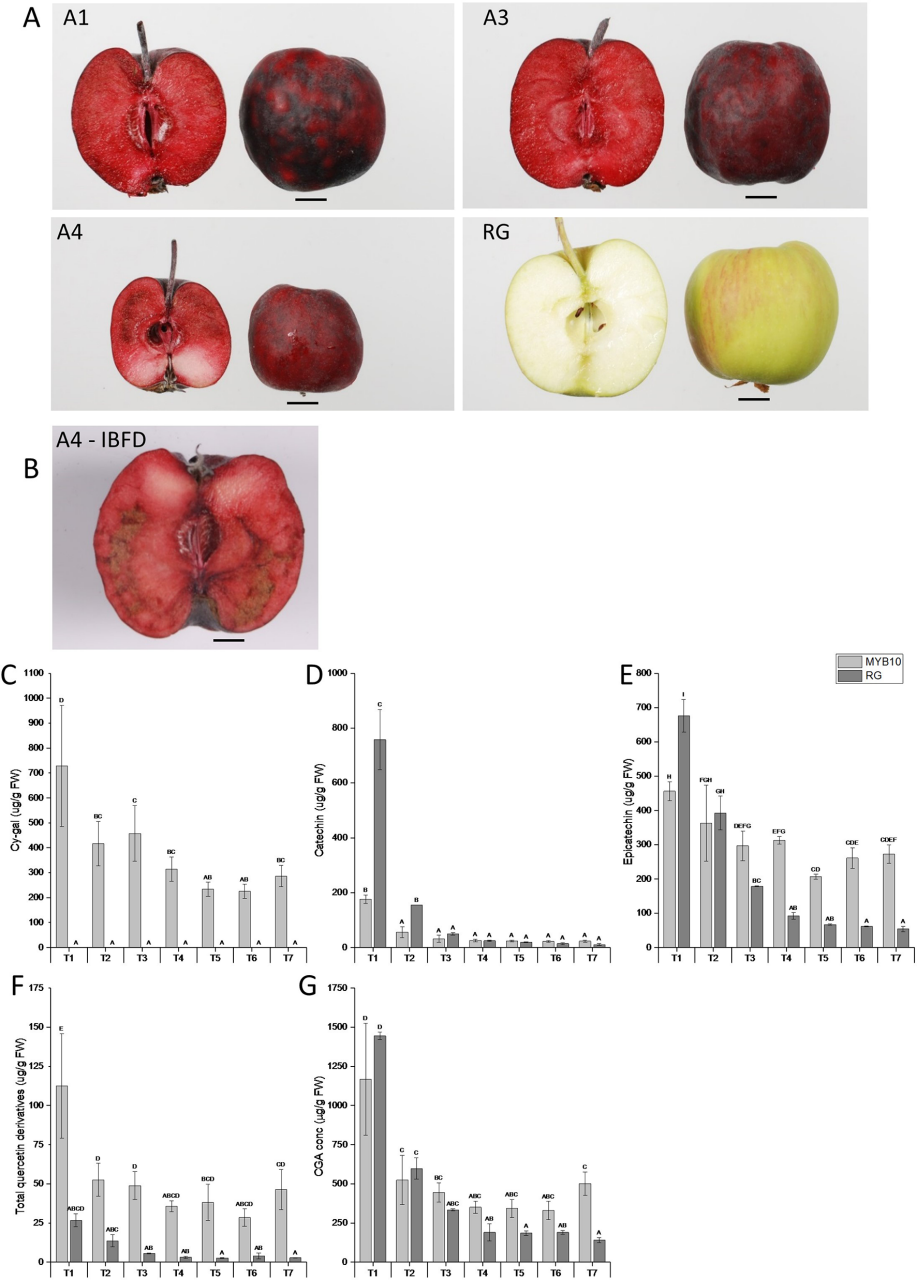
Representative images of **(A)** harvested apple fruit from three independent transgenic lines (A1, A3, and A4) and Wild Type (WT) and **(B)** incidence of IBFD in line A4. Chemical analysis *via* HPLC of metabolites in pooled transgenic lines (MYB10) and WT ‘Royal Gala’ (RG) of **(C)** cyanidin galactoside (Cy-gal) **(D)** catechin **(E)** epicatechin **(F)** total quercetin derivatives and **(G)** chlorogenic acid (CGA). Mean of three biological replicates. T1, 35 Days After Full Bloom (DAFB); T2, 65DAFB; T3, 85 DAFB; T4, 110 DAFB; T5, 120 DAFB; T6, 130 DAFB; and T7, 140 DAFB. Error bars show ± SEM, different letters indicate statistical difference with *P* < 0.05.

‘Royal Gala’ is a cultivar with little reported incidence of IBFD. There are reports of flesh browning but these tend to be after prolonged storage (Lee et al., 2016). We used three independent transgenic lines with high concentrations of flesh anthocyanin to test any link with flesh browning (**Figure 1A**). At harvest (T7), some of the transgenic fruit showed high incidence of IBDF (**Figure 1B**). In WT ‘Royal Gala’ and two of the transgenic lines (A1, A3) there was no IBFD at harvest. However, in the line with the highest anthocyanin content in the flesh, A4, IBDF was apparent in 16% of fruit at harvest (**Figure 1B**). After 10 weeks in standard storage conditions at 0.5°C, the incidence of IBFD was 67, 23, and 60% for the transgenic lines A1, A3, and A4, respectively. No IBFD was detected in any of the ‘Royal Gala’ fruit assessed. All the fruit tested at harvest, including WT, were relatively small compared with field-grown fruit and line A4, in particular, appears smaller than WT (**Table 1**). Both A1 and A3 showed lower fruit firmness than WT. Line A4 also showed a difference in SSC (*P* <0.001).

**Table 1:** Fruit assessment for average weight, firmness and soluble solids content (SSC) of WT and three independent transgenic lines of ‘Royal Gala’ at harvest and after 10 weeks in storage. Incidence of browning by percentage of flesh showing symptoms by visual observation.

Line	Weight (g)	Firmness (kgf)	SSC (°Brix)	IBFD (%) at harvest	IBFD (%) after 10 wks
WT	88.9 ± 9.5	10.5 ± 0.5	11.7 ± 0.4	0	0
A1	68.6 ± 7.7	9.0 ± 0.5	12.2 ± 0.2	0	67
A3	53.9 ± 6.1	9.1 ± 0.5	14.1 ± 0.4	0	23
A4	49.8 ± 5.3	13.1 ± 0.3	11.5 ± 0.3	16	60

Values are averages ± SEM.

### Metabolic Comparison of MYB10 Fruit During Development

Previously it has been shown that the most dramatic effect of over-expression of MYB10 is a greater than a 20-fold increase in total anthocyanins in whole fruit (Espley et al., 2013). This is particularly evident in the fruit flesh, which goes from non-detectable amounts of anthocyanin in ‘Royal Gala’ to more than 700 μg/gFW in the transgenic lines. However, there are also significant changes in other polyphenols and these, together with the anthocyanin, may contribute to a substrate pool. We analysed this in the highly anthocyanic lines under-going IBFD (**Figure 1C**). This confirmed a trend to have higher concentrations of epi-catechin, chlorogenic acid (CGA), and quercetin (**Figures 1D–G**). In contrast, by maturity catechin was not significantly different between control and MYB10 lines (**Figure 1D**). As has been commonly reported, many of the major polyphenols in apple are highly abundant at the earlier stages in fruit development (Henry-Kirk et al., 2012) but the concentrations reduce as the fruit expands and matures. While there is some evidence for this in the anthocyanic transgenic lines, the reduction in these key metabolites is at a lower rate than in ‘Royal Gala’ and relatively high concentrations of epicatechin, CGA and quercetin are maintained in the ripe fruit. Since these polyphenols are a potential source of enzymatic oxidation substrate, we measured the peroxidative potential of fruit throughout the development series.

Enzymatic browning in fruit can occur when phenolic compounds are oxidised, so we measured total peroxidase activity using the rate of conversion of guaiacol to tetraguaiacol. Total peroxidative activity in control ‘Royal Gala’ fruit flesh steadily declined during fruit development to very low rates by maturity (**Figure S1**). In contrast, peroxidative activity was seen to increase in the MYB10 lines.

### Ethylene Induction in MYB10 Lines

**Figure 2:**
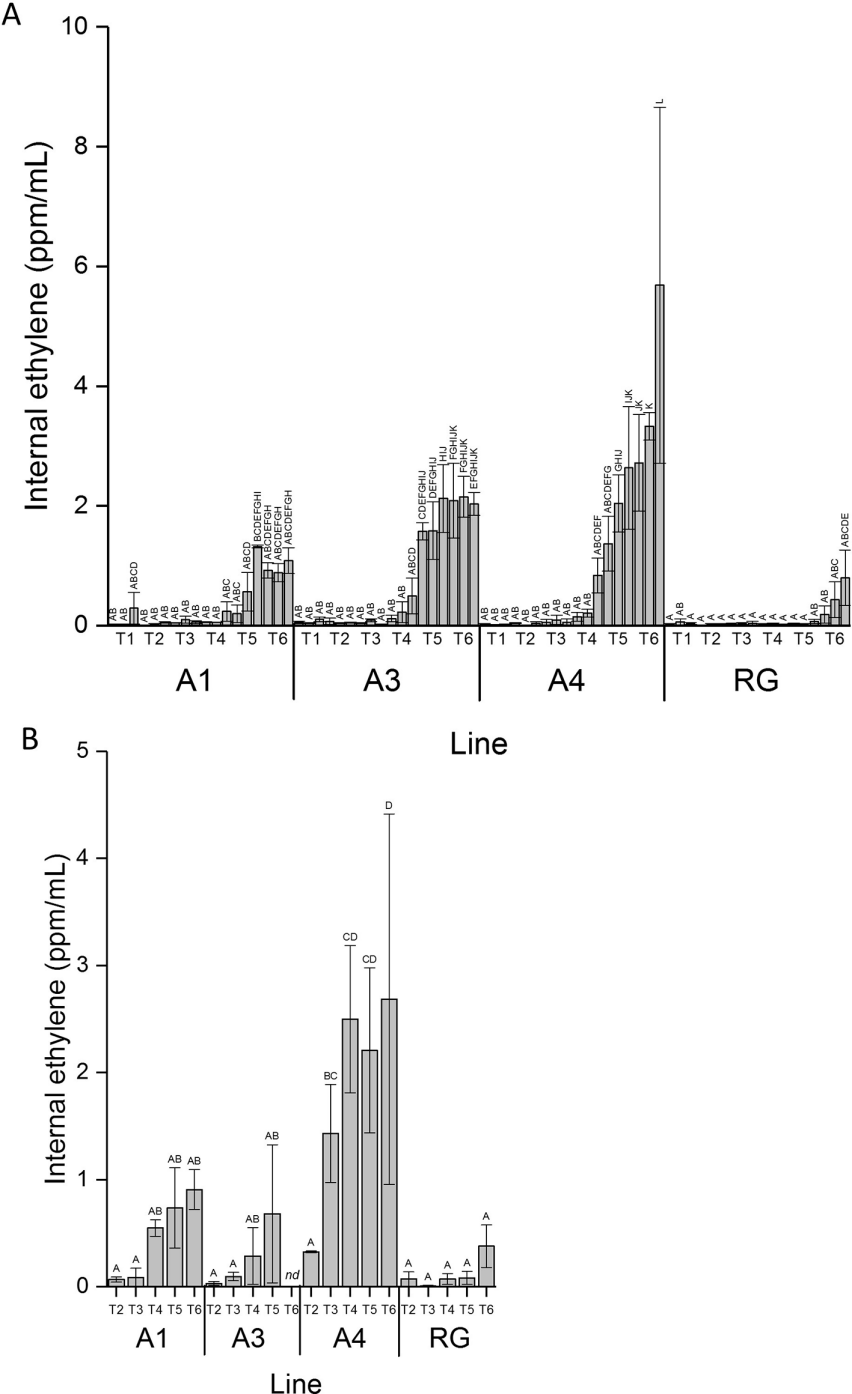
Ethylene induction was earlier in anthocyanic fruit. **(A)** Internal ethylene concentration taken from the same fruit on the tree over fruit development for lines A1, A3, and A4 and WT control (RG). **(B)** Internal ethylene concentration taken from fruit harvested from the tree from time points T2 to T6. Mean of five biological replicates. T1, 35 DAFB; T2, 65DAFB; T3, 85 DAFB; T4, 110 DAFB; T5, 120 DAFB; T6, 130 DAFB. Error bars show ± SEM, different letters indicate statistical difference with *P* < 0.05.

Internal ethylene concentration (IEC) measurements were performed on attached fruit through fruit development using an *in planta* method. A sample of apple fruit core cavity gas was extracted while fruit remained on the tree, by insertion of a needle into the core cavity space. These measurements, made on three individual apples from the three independent lines of MYB10 ‘Royal Gala’, and four individual control ‘Royal Gala’ fruit, were carried out over a 90-day period. Ethylene production for the transgenic lines increased at a much earlier point in fruit development (**Figure 2A**). Low rates of ethylene production occurred in these lines before T4, after which ethylene increased. By T5, IEC in two of the transgenic lines had reached greater than 2 ml/L while in WT it was less than 0.5 ml/L. As expected, ethylene was detected in the WT control fruit commensurate with the onset of on-tree ripening between T5 and T6.

IEC was also assessed destructively with off-tree fruit measurement at six developmental time points equivalent to 65, 85, 95, 100, and 108 DAFB on three separate biological replicates for each line and controls (**Figure 2B**). These standard assays confirmed results for the on-tree assessments with an early ethylene production detected in all transgenic lines compared with the control fruit.

### Effects on Starch Concentration

Flesh starch concentration in transgenic MYB10 apple fruit was significantly lower than in WT ‘Royal Gala’ fruit (ANOVA main effect of wild type over transgenic lines; *P* < 0.0001) (**Figure S2**). Differences were evident from developmental stage T2 and were maintained throughout fruit growth. Starch degradation is an indicator of apple fruit maturity (Brookfield et al., 1997). Minimal starch concentrations at T6 and T7 indicated that fruit maturation was near completion in transgenic fruit at T6, whilst in ‘Royal Gala’ WT fruit starch degradation was still occurring by T7 stage. Furthermore, the rate of starch degradation was faster in transgenic MYB10 apple fruit compared to the WT (**Figure S3A**). Flesh sorbitol concentration in transgenic MYB10 apple fruit was significantly higher than in WT ‘Royal Gala’ fruit (ANOVA main effect of transgenic lines over wild type; *P* < 0.001) (**Figure S2B**). Fructose concentration was higher in WT ‘Royal Gala’ fruit than that in the transgenic fruit (ANOVA main effect of wild type over transgenic lines; *P* < 0.001) (**Figure S3B**). Fruit sucrose concentration was significantly higher in WT ‘Royal Gala’ than the transgenic lines at T7 (*P* < 0.05; **Figure S3C**). Glucose concentration was overall higher in transgenic lines, although the differences were not statistically significant (**Figure S3D**).

### Expression Analysis of PPO and Ethylene-Related Genes

**Figure 3:**
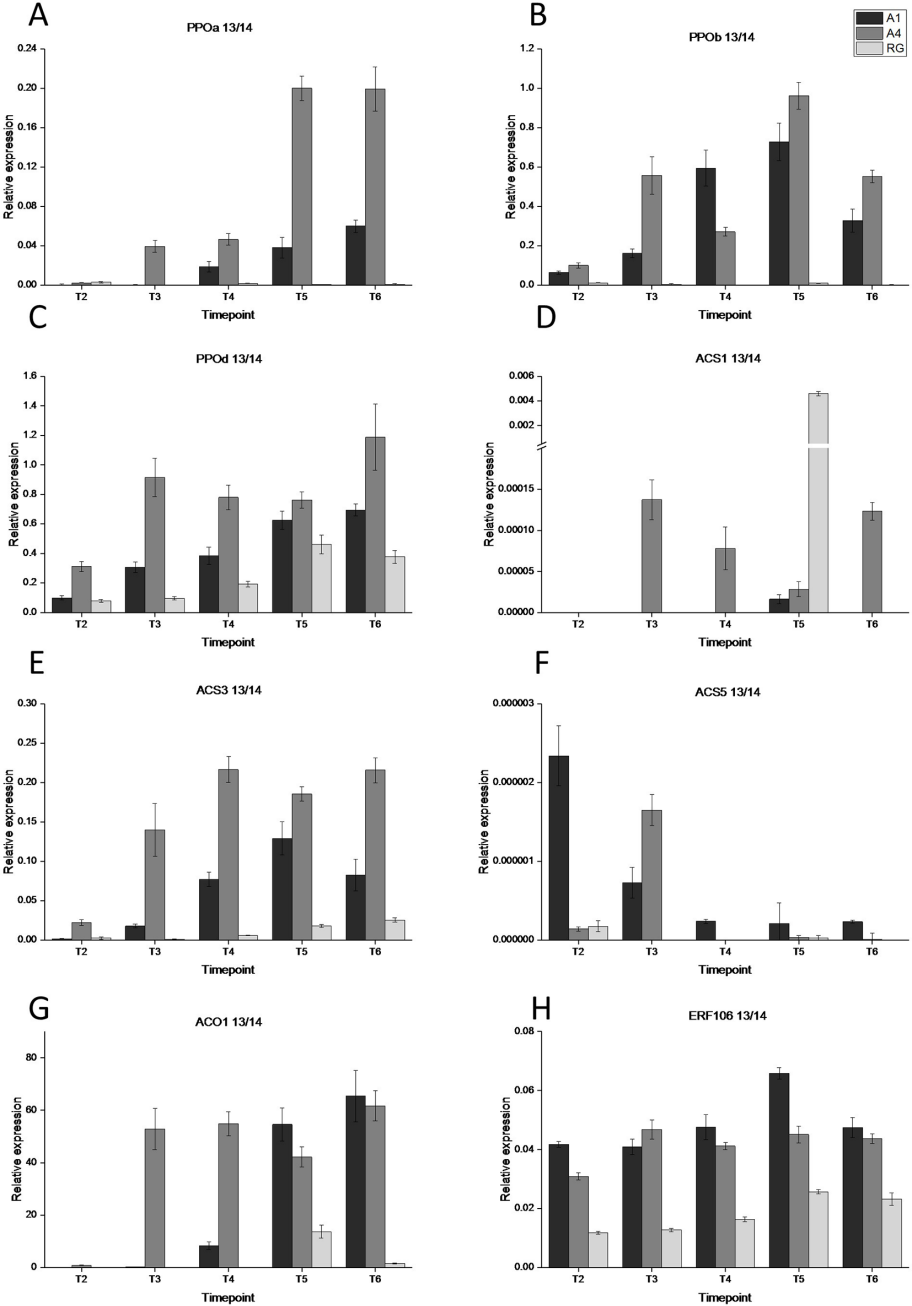
Gene expression analysis of eight maturity and browning associated genes at five time points during fruit development during 2013-2014. **(A)** PPOa, **(B)** PPOb, **(C)** PPOd, **(D)** ACS1, **(E)** ACS3, **(F)** ACS5, **(G)** ACO1, and **(H)** ERF106. T1, 35 DAFB; T2, 65DAFB; T3, 85 DAFB; T4, 110 DAFB; T5, 120 DAFB; T6, 130 DAFB. Expression was normalised to EF1a and error bars are SE of four technical replicates of at least 5 pooled fruit per time point. Expression is shown for three lines; dark grey—A1, medium grey—A4, and light grey—RG.

We tested a number of PPO genes (PPOa, PPOb, PPOc, and PPOd) for transcript abundance in both red and white apple flesh tissue. There were high rates of expression in both transgenic lines (A1 and A4) compared with WT control for all tested genes (no transcript was detected for PPOc) (**Figure 3**). This expression was evident at early time points in fruit development, T2 for PPOb and PPOd and T3 for PPOa in the transgenic lines. In WT ‘Royal Gala’ there was no detectable expression in PPOa and PPOb and a gradual increase in expression of PPOd, albeit at a lower rate than the red-fleshed lines. In most cases line A4, the most highly pigmented and prone to IBFD, showed the highest expression for all the genes tested.

With evidence for an early peak in ethylene production in the anthocyanic fruit, the key genes in the ethylene biosynthesis pathway, ACO and ACS, were tested for transcript abundance in the fruit flesh of two transgenic lines (A1 and A4) and WT (**Figure 3**). For ACO1, transcript was detectable in both A1 and A4 at T4, while in ‘Royal Gala’ WT transcript was not detected until T5. The highest ACO transcript abundance was detected in the A1 and A4 transgenic lines at the final time point, T6. For ACS1, transcript was at low levels with an anomalous peak of expression for RG at T5. For the previous year tested, an early peak of ACS1 expression was evident for both transgenic lines tested at T4 but at the final time point the highest expression was seen in control tissue (**Figure S4**). For ACS3, the most abundant ASC gene, transcript was detected at an even earlier stage (T3) for A1 and A4 but was evident in all samples, including WT control, at T4. By the final time point (T6), expression was higher in the transgenic lines. ACS5 was never detected in control samples but there was low expression in A1 and greater expression, albeit at a low level, in A4.

**Figure 4:**
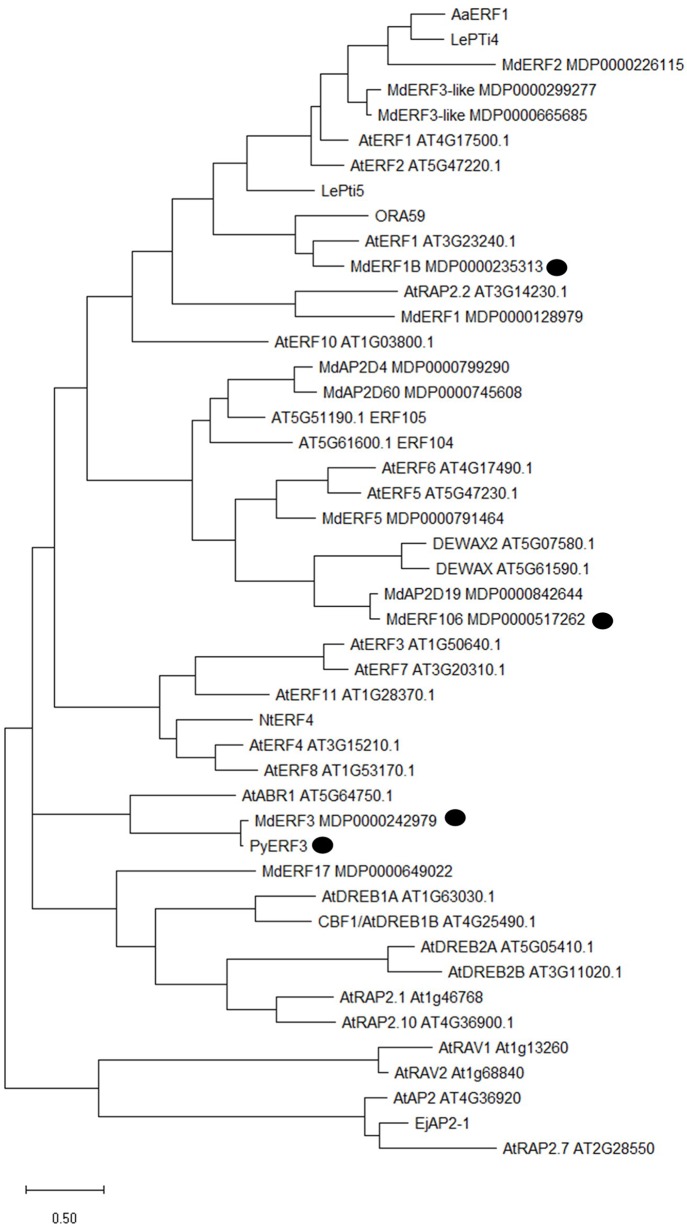
Phylogenetic tree analysis of MdERF106 protein and other related AP2/ERF TFs from different species. Published Rosaceous proteins implicated in ethylene responses are indicated by a black circle. Protein identifiers are either published MDP numbers for *Malus*, TAIR for *Arabidopsis*, or NCBI accession numbers for other species as follows: LePti4 (NM_001347076.1), LePti5 (U89256.1), ORA59 (NM_100497.3), AaERF1 (JN162091.1), PyERF3 (MF489220), NtERF4 (NM_001325253.1), and EjAP2-1 (KM506584.1).

Based on data from a previous study using RNA-sequencing on red and white fleshed apples (Wang et al., 2018), we selected ERF106 as a possible candidate gene for involvement in the regulation of anthocyanin-related early ethylene production. We found low levels of transcript abundance throughout fruit development in ‘Royal Gala’ but consistently higher abundance in both of the transgenic lines tested. MdERF106 is similar to Arabidopsis AtDEWAX and AtDEWAX2 (AtERF107/ERF106) (**Figure 4**, **Figure S5**) which negatively regulate cuticular wax biosynthesis (Go et al., 2014; Kim et al., 2018), and other ERFs such as AtERF5 and AtERF6 involved in biotic and abiotic stress response (Moffat et al., 2012; Dubois et al., 2013). Previously it has been shown that MYB1/10 activates expression of apple ERF3 (a close homologue to pear ERF3) to increase ethylene emission from apple callus (An et al., 2018), while MdERF1B has been shown to alter anthocyanin and proanthocyanidin concentration (Zhang et al., 2018). In pear PyERF3 interacts with PyMYB114 to co-regulate anthocyanin biosynthesis (Yao et al., 2017). These ERFs are less similar to MdERF106.

### Activation of Non-Anthocyanin Related Genes by MYB10

**Figure 5:**
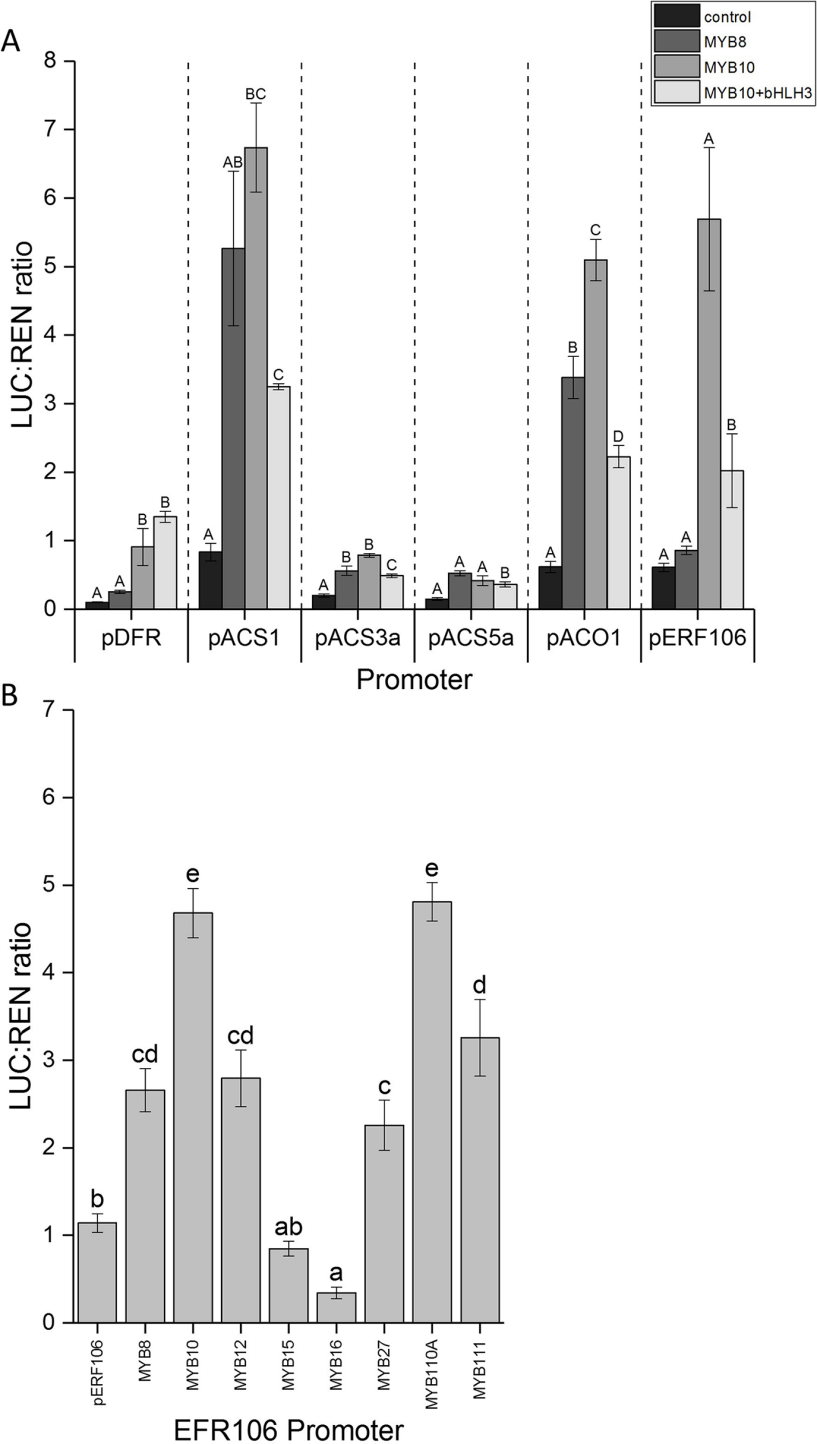
**(A)** MYB10 activates the promoters of ethylene-associated genes. The promoter sequences from ACS1, ACS3, ACS5, ACO1, and ERF106 were isolated and cloned into the dual luciferase reporter construct and infiltrated into *N. benthamiania* leaves in combination with MYB10 with and without bHLH3. MYB8 was included as a control. **(B)** Anthocyanin activating MYBs activate the promoter of ERF106. The promoter sequence from ERF106 was isolated and cloned into the dual luciferase reporter construct and infiltrated into *N. benthamiania* leaves either on its own or in combination with activating or repressing MYB TFs. Different letters indicate statistical difference with *P* < 0.05 (7A refers to each promoter independently). Mean of four biological replicates. Error bars show ± SEM.

To test for the possibility of direct activation of ethylene-related gene by MYB10, we cloned the promoters of ACO, ACS1, ACS3, ACS5, and ERF106, which were fused with the luciferase reporter gene sequence. These were then tested for MYB10 activation using the dual luciferase assay. Since MYB10 is known to partner with bHLH3 in the MBW complex and is required for full activation of the anthocyanin structural genes, the apple bHLH3 TF was also included. A non-anthocyanin related MYB, MYB8, was used as a control MYB to test for specificity of MYB10. The promoter fragment of apple DFR was used as a positive control for MYB10 activation. As expected, MYB8 had little activation potential on DFR, while MYB10 did induce promoter activity and luciferase production (**Figure 5A**). This was further enhanced by the co-infiltration of bHLH3.

For the ACS3 and ACS5 promoters there was no apparent activation by MYB10, with or without bHLH3, or for the control MYB8. However, both MYB8 and MYB10 appeared to activate ACS1, suggesting a non-specific MYB activation. This was somewhat reduced for MYB10 when co-infiltrated with bHLH3. For ACO1, there was also activation by MYB8 and MYB10, with higher activation for the latter that was then somewhat diminished by the inclusion of bHLH3.

We tested the MYBs for activity on the ERF106 promoter fragment. Here we found no detectable activation by the control MYB8 but a strong activation by MYB10. Again, this was reduced with the co-infiltration of bHLH3.

To further test the specificity of MYB10 on the ERF106 promoter, a number of known apple flavonoid activating and repressing MYB TFs were assayed against the ERF106 promoter. The strongest activation was seen with the anthocyanin-related activators MYB10 and MYB110 (**Figure 5B**). A lower level of activation was seen with infiltration of the flavonoid-related MYB12, while the anthocyanin repressors showed mixed results, with no activation by MYBs 15 and 16 but some activation by both MYB27 and MYB111.

## Discussion

Of the widely cultivated varieties of apple, ‘Royal Gala’ does not normally exhibit IBFD. However, the MYB10-induced ectopic accumulation of anthocyanin in fruit flesh leads to a severe browning phenotype, suggestive of a link between flesh colour and browning. Alternatively, an increase in other polyphenols may be the cause, providing increased substrate for downstream oxidation. The concentration of polyphenols in apple fruit usually reduces over development but in MYB10 lines a relatively high concentration of epi-catechin, CGA, and quercetin is still present at maturity, in addition to the high concentration of cyanidin-galactoside. The incidence of IBFD in these highly anthocyanic transgenic lines is resonant with data from a traditionally bred red-fleshed apple population (Volz et al., 2013). In the red-fleshed breeding populations tested, quantitative trait loci (QTL) for IBFD were detected at two genetic locations on Linkage Group (LG) 9 and LG6, consistent with a QTL for red flesh, and suggesting a strong genetic link between MYB10 and IBFD.

Adding to these possibilities, there is also an increase in the ripening-related gaseous hormone, ethylene. Since the developmental accumulation of anthocyanin colour in apple is part of the ripening process, it is likely that these processes are connected. Here we show that MYB10 may play a role in ethylene biosynthesis, *via* ethylene-related transcription factors and that this premature ethylene production is closely associated with a reduction in fruit quality, ultimately leading to IBDF. A previous study in a F_1_ population of red and white fleshed apples showed a higher concentration in flavonoid content as well as anthocyanin in red fleshed apples (Wang et al., 2015). In this study a comparative transcriptome showed a higher incidence of abiotic and biotic stress-related genes in red flesh, and that this was associated with an increased production of flavonoids. Whether these stress-related genes are up-regulated in direct response to flavonoid production or the potential detrimental effects of an increase in flavonoids leading to premature ripening is yet to be fully determined. This study was conducted on ripe fruit although some ethylene-related genes such as ACO (gene ID 103404960, equivalent to MDP0000195885) showed greater transcript abundance in red flesh compared to white.

### Anthocyanin Is Associated With Major Changes in Fruit Physiology

The ectopic accumulation of anthocyanin does not necessarily lead to reduced fruit quality. This is convincingly demonstrated in tomato where elevation of anthocyanin in transgenic purple tomato fruit leads to an extended shelf life and a reduction in susceptibility to grey mould (Zhang et al., 2013). In this study, it appears that the increased anthocyanin boosts antioxidant capacity in the fruit which is likely to slow the over-ripening process. This is in direct contrast to results presented here for apple. We have previously shown an elevated antioxidant capacity for these highly anthocyanic apples (Espley et al., 2014) despite a reduction in ascorbic acid, suggesting that the increase in anthocyanins and flavonoids are the reason for this increase. However, this increased capacity did not improve shelf life and the red-flesh apples over-ripened more quickly than the white-fleshed controls. Like apple, tomato is a climacteric fruit and so reliant on ethylene for ripening. In the purple tomatoes, ethylene production was 2-fold greater than control fruit, although this was just after the breaker stage. This differs for apple, where we see an ethylene burst earlier in fruit development.

There are reports of flesh browning (flesh breakdown) in ‘Royal Gala’ but these tend to be after prolonged storage of more than three months and may be more prevalent in larger fruit (Lee et al., 2013; Lee et al., 2016). In these transgenic lines, a high incidence of IBFD is visible at (and even before) harvest (Line A4) and is severe by 10 weeks in storage, while WT ‘Royal Gala’ showed no visible symptoms.

Apple has an unusual carbohydrate metabolism compared to the more commonly studied model plants (e.g. Arabidopsis) as sucrose is not the major carbohydrate metabolite. Indeed sorbitol is the major photosynthetic product in apple leaves (60–80% of sugars), and it is translocated in the phloem to sink tissues where it is quickly converted into fructose by sorbitol dehydrogenase (Loescher, 1987; Klages et al., 2001; Wu et al., 2015), so that sorbitol concentrations in fruit are usually low (Yamaki and Ishikawa, 1986). Apple fruit are mainly fructose accumulators, although early in development they store both fructose and transitory starch in similar amounts (Li et al., 2018). Fruit sorbitol dehydrogenase genes respond to variable levels of source sorbitol, decreasing in expression when leaf sorbitol levels decrease. In sorbitol dehydrogenase antisense lines, translocated sorbitol is reduced, lowering the levels of sorbitol in fruit (Teo et al., 2006; Li et al., 2018). In our experiment, transgenic MYB10 lines had fruit with higher a concentration of sorbitol in the fruit flesh than the wild type ‘Royal Gala’. This phenotype could be due to the up-regulation of leaf sorbitol metabolism in transgenic MYB10 lines or by a down-regulation of the sorbitol dehydrogenase genes in the fruit so that sorbitol is converted more slowly into fructose. However, it could be also linked to the maturation stage the fruit have reached. Onset of net transitory starch degradation in apple occurs whilst fruit are still on the tree (Zhang et al., 2017), and it is an indication of fruit maturation (Brookfield et al., 1997). In apple, starch degradation is a process with low dependency but high sensitivity to ethylene (Johnston et al., 2009) which means an earlier onset of endogenous production of the hormone (from T3 rather than T5 in WT) could trigger faster starch degradation in the fruit, as observed in this study with the transgenic MYB10 lines. Sorbitol is likely to be linked to the ripening stage (Aprea et al., 2017). The accumulation of sorbitol in the MYB10 lines could be a result of the fruit switching to a ripening stage at T4–T5, and sorbitol translocated to the fruit is converted to fructose more slowly.

In the highly anthocyanic MYB10 fruit, there is an increase in a number of polyphenols as previously shown (Espley et al., 2013). These polyphenols could provide added substrate to fruit flesh for enzymatic browning to occur. The peroxidative activity is also higher in all the MYB10 lines, therefore, both the potential substrates for browning and the peroxidative enzymes are at greater concentration in red-fleshed lines than in WT control. This is further shown in the PPO expression analysis, where an increase in transcript in both transgenic lines tested was evident for PPOa, PPOb, and PPOd. Results from the published transcriptomic analysis of red versus white apple flesh at harvest (Wang et al., 2015), shows that PPOa, and particularly PPOb, are more highly expressed in the red flesh while PPOc and PPOd are more highly expressed in the white fleshed fruit. It would appear that the key finding presented in our data is the demonstration of very early PPO expression.

### Early Production of Ethylene *via* the Up-Regulation of Ethylene Genes in Red-Fleshed Fruit

The rate of ethylene production can vary widely between apple cultivars (Harada et al., 2000). It has also been previously shown that mature red-fleshed apples start to produce more ethylene than those with white flesh just two days after harvest (An et al., 2018). In our study, apples from the same genetic background were used, so from a genetic level it might be expected that ethylene production would be similar. However, we detected a very different ethylene production profile for all three transgenic lines compared with WT. Production was at levels normally consistent with the onset of the ripening-related ethylene burst but at four to six weeks earlier than expected. The collection of ethylene samples while fruit were still attached to the tree was chosen as the most representative method of assessing the actual increase in ethylene production in developing fruit. As this is method is novel, we also used a standard practice for ethylene assessment for harvested fruit and found very similar patterns.

Studies have shown that the biosynthesis of ethylene is transcriptionally regulated (Schaffer et al., 2013). ACC synthase (ACS) converts *S*-adenosyl-L-methionine to 1-aminocyclopropane-1-carboxylate (ACC) and is thought to be the rate limiting step (Yang and Hoffman, 1984). We tested the transcript abundance of three versions of ACS: ACS1, ACS3, and ACS5. For all three versions we found an increase in transcription at a considerably advanced stage in the anthocyanic fruit compared to WT ‘Royal Gala’. ACS1 is associated with the ripening-specific climacteric burst (Harada et al., 2000), while ACS3 is transcriptionally active before ACS1 and is likely to be involved in the transition from system-1 to system-2 ethylene biosynthesis (Wang et al., 2009). ACS3 transcript was just detectable in the transgenic lines at T3, and clearly detectable at T4, as was ACS1. However, there did not appear to be negative feedback for ACS3, as previously reported (Wang et al., 2009) and transcription increased as fruit developed. ACS5 is possibly more associated with wounding rather than ethylene-related ripening (Costa et al., 2005). The transcript abundance of ACS5 was detectable at low levels in the MYB10 lines, particularly A4, but was at very low rates.

The oxidation step performed by ACC oxidase (ACO) to form ethylene follows the ACS-mediated conversion to ACC. ACO transcript abundance was measured and the first major peak in expression was seen at T4 but only in the transgenic lines A1 and A4. At T5 transcript was also detected in control fruit. There are other ACO gene family members (not tested here) that may also contribute to the rise in ethylene and ACO has been linked to internal browning in white-fleshed fruit (Mellidou et al., 2014). The pattern for the highly anthocyanic fruit shows that the presence of ACS3 transcript at T3 precedes both ACS1 and ACO transcript at T4 while the later expression of ACS3 in control fruit at T4 ACO precedes ACO1 at T5 and ACS1 at T6. Since ACS1 has been shown to be responsible for system-2 type autocatalytic ethylene production (Tan et al., 2013) the results here are entirely consistent with premature ripening in MYB10 fruit.

### A Proposed Model for the Association Between Anthocyanin and IBFD

The Ethylene Signalling Cascade Ends With the ERFs and These Can Either Activate or Repress Ethylene production as well as regulating the expression of ripening-related genes (Liu et al., 2015). In apple, the ERF2 TF has been shown to negatively regulate ethylene biosynthesis by suppressing the transcription of ACS1 (Li et al., 2016). Previous data has shown that one ethylene-related TF, ERF106, was differentially expressed in red compared with white apple flesh (Wang et al., 2018).

A study by Zhang et al. (2018) showed that ERF1B was capable of interacting with the promotors of anthocyanin and proanthocyanidin MYB TFs. This interaction leads to increases in the related metabolites and suggests that ethylene regulation and anthocyanin regulation might be linked in either direction.

It appears that both MYB apple flesh anthocyanin regulators, the Type 1 MYB10 and Type 2 MYB110 (Chagné et al., 2013) were capable of activating the promoter of ERF106. Some of the other MYBs tested did demonstrate some level of activation, but to a lesser extent. The MYB binding sites may confer some non-specific binding and hence, activation. Interestingly, two known apple anthocyanin repressors, MYBs 15 and 16 (Lin-Wang et al., 2011), showed no activation with reporter levels below that of the control. Further work is required to test if these MYB repressors can compete with activators or repress promoter activity.

**Figure 6:**
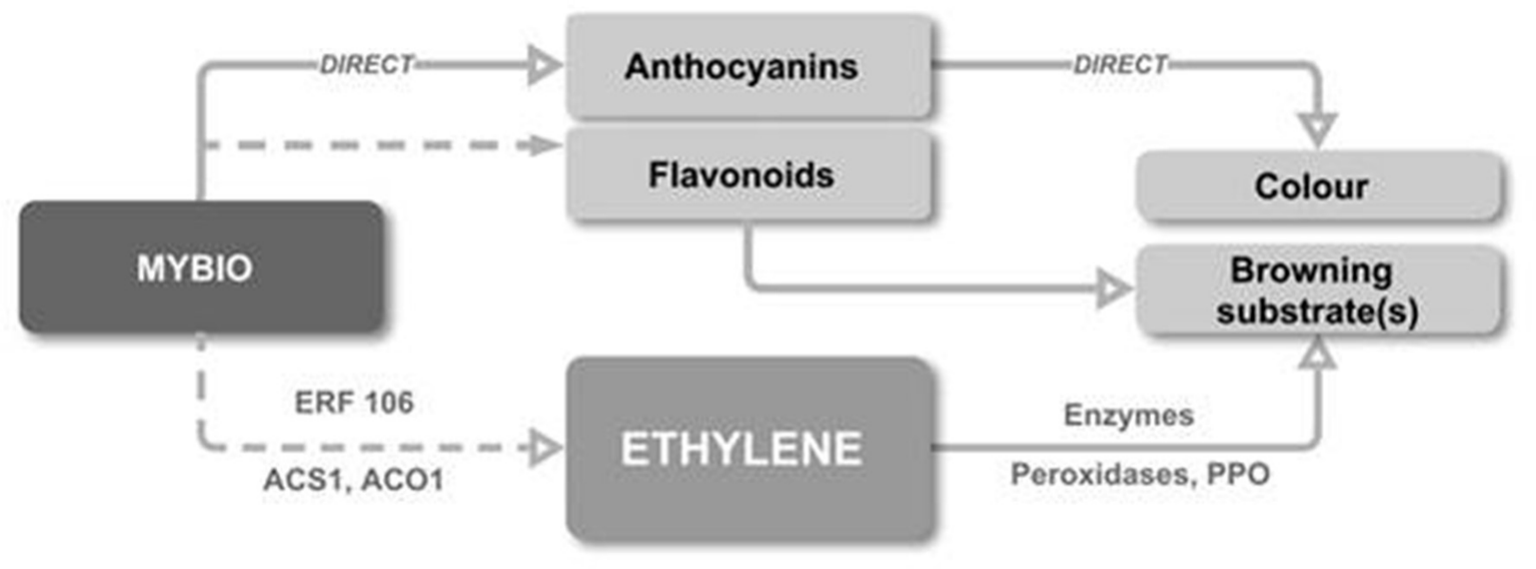
A proposed model for the link between elevated flesh anthocyanin and IBFD. The up-regulation of MYB10 directly increases anthocyanin concentration and indirectly causes increased production of flavonoid. MYB10 is also associated the early onset of ethylene production, possibly *via* interaction with ERF106. Ethylene production advances fruit maturity and an increase in transcription and enzyme activity of PPO. These events lead to early ripening, the production of peroxidise enzymes and a pool of additional substrate which culminate in IBFD.

We propose a model (**Figure 6**) whereby the up-regulation of MYB10 directly increases anthocyanin concentration by regulating the anthocyanin biosynthetic pathway and indirectly causes the increased production of flavonoids by enhanced pathway flux. MYB10 is also associated with an increase in early ethylene production, possibly *via* interaction with ERF106. This ethylene production advances mechanisms that drive fruit maturity, including the elevation of PPO. These direct and indirect events produce a scenario where early ripening is triggered and where the production of peroxidise enzymes and large pool of additional substrate come together to culminate in IBFD.

### Summary

The data presented here describes a mechanism for elevated IBFD in anthocyanin tissue in apple fruit. This has implications for the breeding of high quality novel red-fleshed cultivars. The genetic background may be key to IBDF evasion, such as the use of low ethylene producing parents in a breeding population. Alternative strategies to avoid IBDF could include genetic manipulation such as demonstrated by knocking out PPO in Arctic^®^ apple (Waltz, 2015) which could also be achieved using CRISPR/Cas9 gene editing approach.

## Data Availability Statement

All datasets for this study are included in the manuscript/**Supplementary Files**.

## Author Contributions

RE, DL, BP, RH-K, MH, and SO’D contributed to the harvest and postharvest assessments, qPCR, transactivation experiments. MP and BP conducted peroxidase assays. HB and SN performed carbohydrate assessment. TM performed chemical analysis. RE, AA, RV, and JJ developed the experimental design. RE, AA, SN, and BP wrote the manuscript and all authors contributed to editing.

## Conflict of Interest

The authors declare that the research was conducted in the absence of any commercial or financial relationships that could be construed as a potential conflict of interest.
