# Supplementary material for: Red to Brown: An Elevated Anthocyanic Response in Apple Drives Ethylene to Advance Maturity and Fruit Flesh Browning
Source: Front Plant Sci. 2019 Oct 9;10:1248. doi: 10.3389/fpls.2019.01248 (PMC6794385; doi:10.3389/fpls.2019.01248)
Supplement: Supplementary file 1 [file Presentation_1.pptx]

## Slide 1
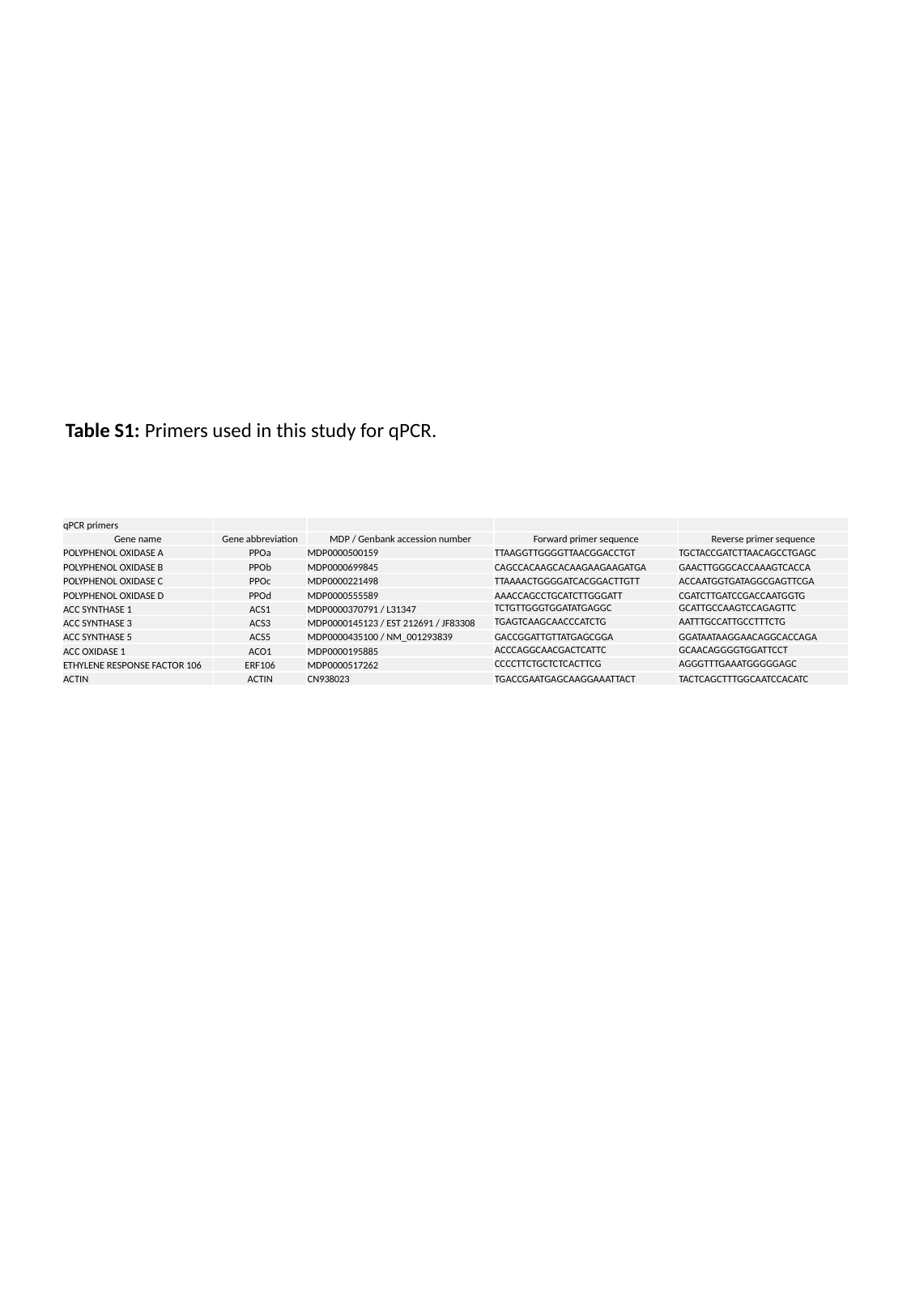

Table S1: Primers used in this study for qPCR.
| qPCR primers | | | | |
| --- | --- | --- | --- | --- |
| Gene name | Gene abbreviation | MDP / Genbank accession number | Forward primer sequence | Reverse primer sequence |
| POLYPHENOL OXIDASE A | PPOa | MDP0000500159 | TTAAGGTTGGGGTTAACGGACCTGT | TGCTACCGATCTTAACAGCCTGAGC |
| POLYPHENOL OXIDASE B | PPOb | MDP0000699845 | CAGCCACAAGCACAAGAAGAAGATGA | GAACTTGGGCACCAAAGTCACCA |
| POLYPHENOL OXIDASE C | PPOc | MDP0000221498 | TTAAAACTGGGGATCACGGACTTGTT | ACCAATGGTGATAGGCGAGTTCGA |
| POLYPHENOL OXIDASE D | PPOd | MDP0000555589 | AAACCAGCCTGCATCTTGGGATT | CGATCTTGATCCGACCAATGGTG |
| ACC SYNTHASE 1 | ACS1 | MDP0000370791 / L31347 | TCTGTTGGGTGGATATGAGGC | GCATTGCCAAGTCCAGAGTTC |
| ACC SYNTHASE 3 | ACS3 | MDP0000145123 / EST 212691 / JF83308 | TGAGTCAAGCAACCCATCTG | AATTTGCCATTGCCTTTCTG |
| ACC SYNTHASE 5 | ACS5 | MDP0000435100 / NM\_001293839 | GACCGGATTGTTATGAGCGGA | GGATAATAAGGAACAGGCACCAGA |
| ACC OXIDASE 1 | ACO1 | MDP0000195885 | ACCCAGGCAACGACTCATTC | GCAACAGGGGTGGATTCCT |
| ETHYLENE RESPONSE FACTOR 106 | ERF106 | MDP0000517262 | CCCCTTCTGCTCTCACTTCG | AGGGTTTGAAATGGGGGAGC |
| ACTIN | ACTIN | CN938023 | TGACCGAATGAGCAAGGAAATTACT | TACTCAGCTTTGGCAATCCACATC |

## Slide 2
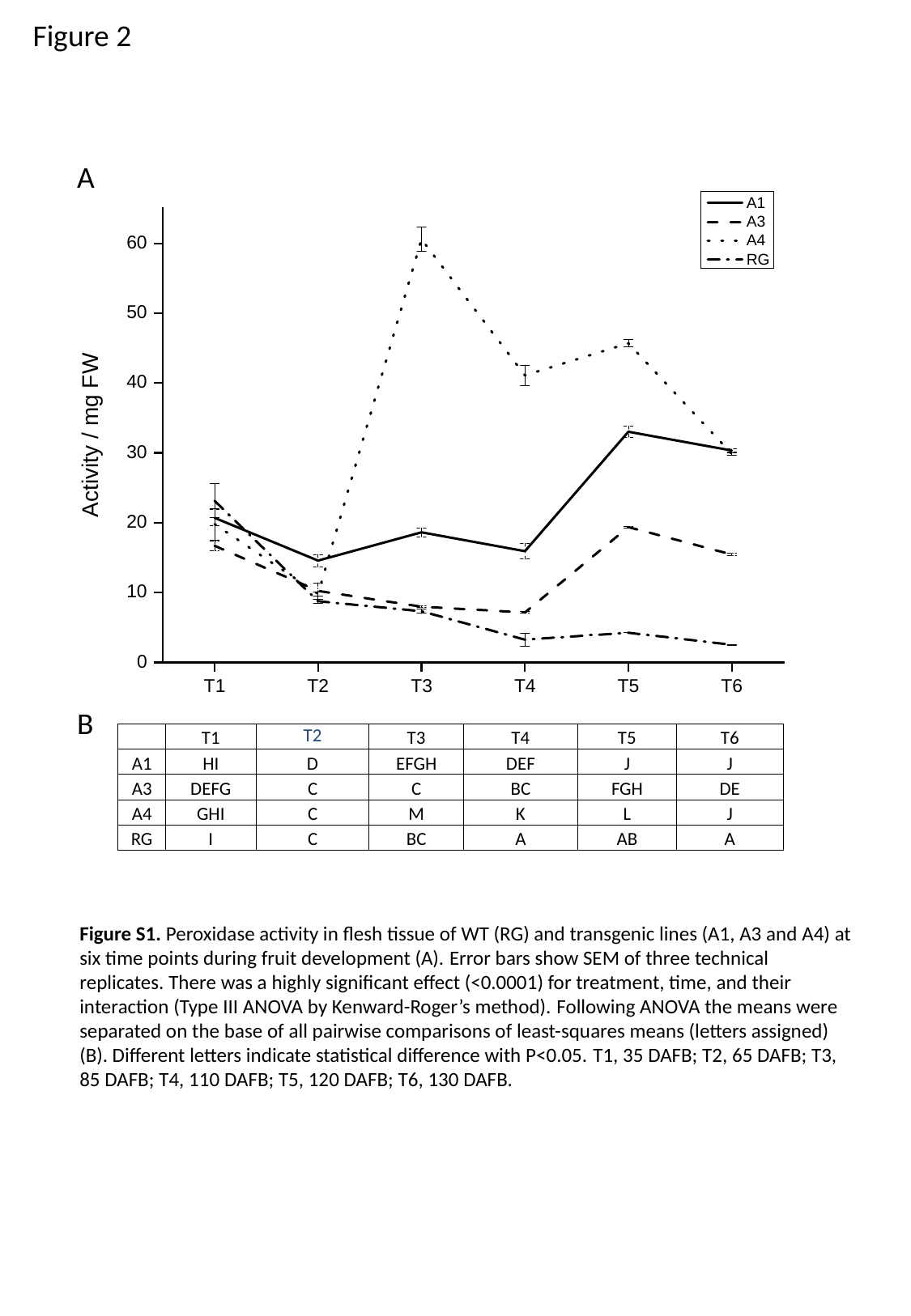

Figure 2
A
B
| | T1 | T2 | T3 | T4 | T5 | T6 |
| --- | --- | --- | --- | --- | --- | --- |
| A1 | HI | D | EFGH | DEF | J | J |
| A3 | DEFG | C | C | BC | FGH | DE |
| A4 | GHI | C | M | K | L | J |
| RG | I | C | BC | A | AB | A |
Figure S1. Peroxidase activity in flesh tissue of WT (RG) and transgenic lines (A1, A3 and A4) at six time points during fruit development (A). Error bars show SEM of three technical replicates. There was a highly significant effect (<0.0001) for treatment, time, and their interaction (Type III ANOVA by Kenward-Roger’s method). Following ANOVA the means were separated on the base of all pairwise comparisons of least-squares means (letters assigned) (B). Different letters indicate statistical difference with P<0.05. T1, 35 DAFB; T2, 65 DAFB; T3, 85 DAFB; T4, 110 DAFB; T5, 120 DAFB; T6, 130 DAFB.

## Slide 3
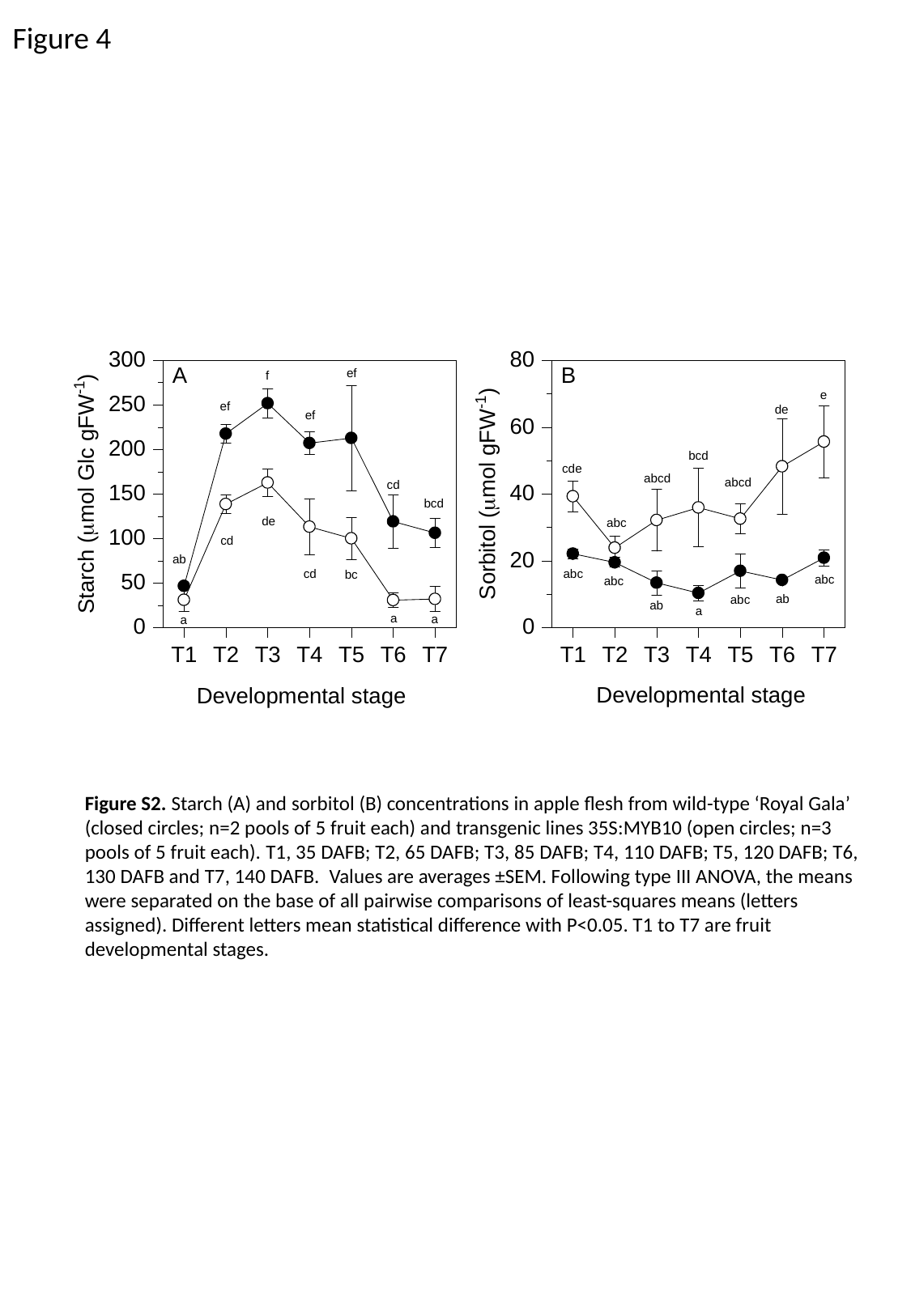

Figure 4
Figure S2. Starch (A) and sorbitol (B) concentrations in apple flesh from wild-type ‘Royal Gala’ (closed circles; n=2 pools of 5 fruit each) and transgenic lines 35S:MYB10 (open circles; n=3 pools of 5 fruit each). T1, 35 DAFB; T2, 65 DAFB; T3, 85 DAFB; T4, 110 DAFB; T5, 120 DAFB; T6, 130 DAFB and T7, 140 DAFB. Values are averages ±SEM. Following type III ANOVA, the means were separated on the base of all pairwise comparisons of least-squares means (letters assigned). Different letters mean statistical difference with P<0.05. T1 to T7 are fruit developmental stages.

## Slide 4
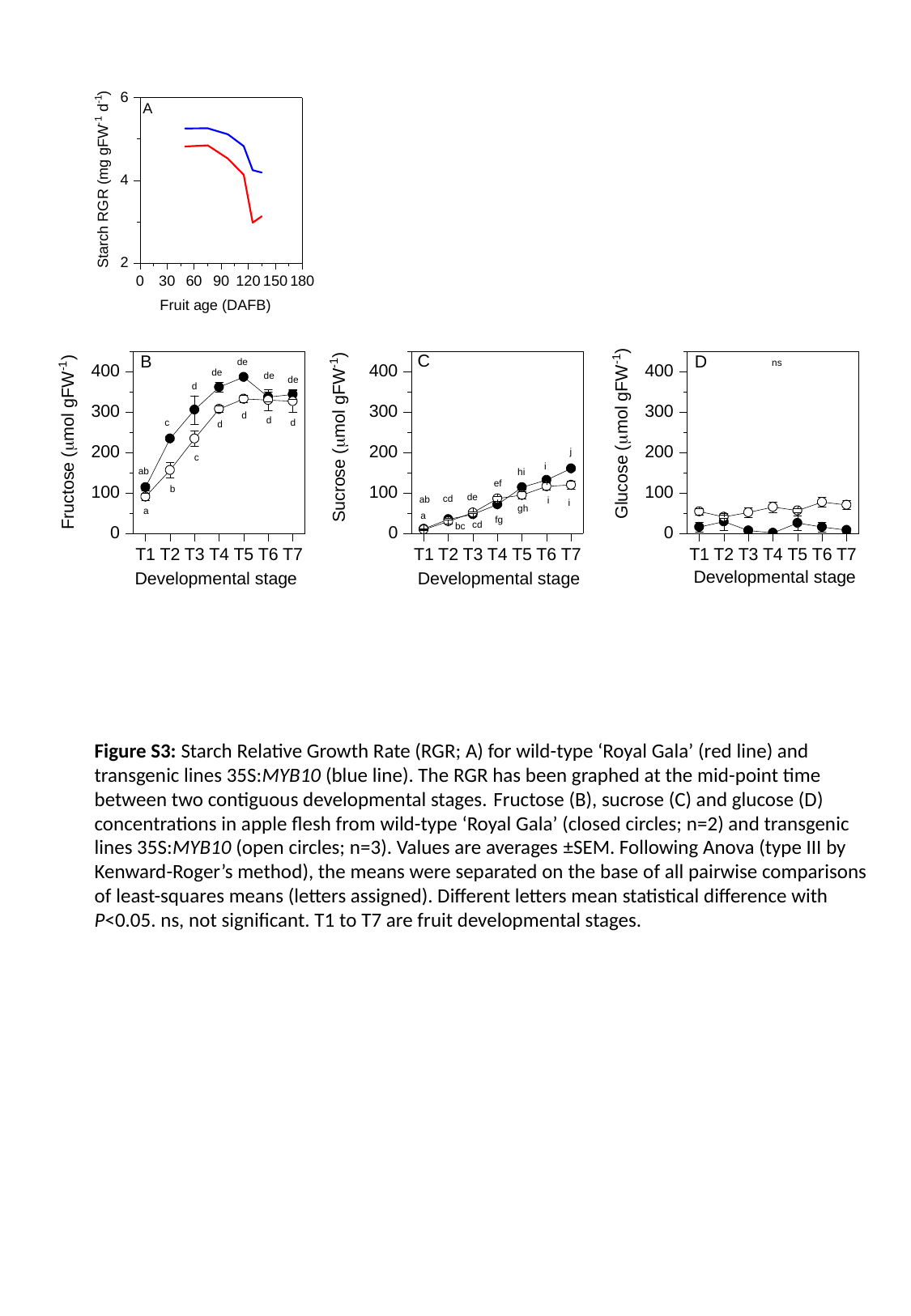

Figure S3: Starch Relative Growth Rate (RGR; A) for wild-type ‘Royal Gala’ (red line) and transgenic lines 35S:MYB10 (blue line). The RGR has been graphed at the mid-point time between two contiguous developmental stages. Fructose (B), sucrose (C) and glucose (D) concentrations in apple flesh from wild-type ‘Royal Gala’ (closed circles; n=2) and transgenic lines 35S:MYB10 (open circles; n=3). Values are averages ±SEM. Following Anova (type III by Kenward-Roger’s method), the means were separated on the base of all pairwise comparisons of least-squares means (letters assigned). Different letters mean statistical difference with P<0.05. ns, not significant. T1 to T7 are fruit developmental stages.

## Slide 5
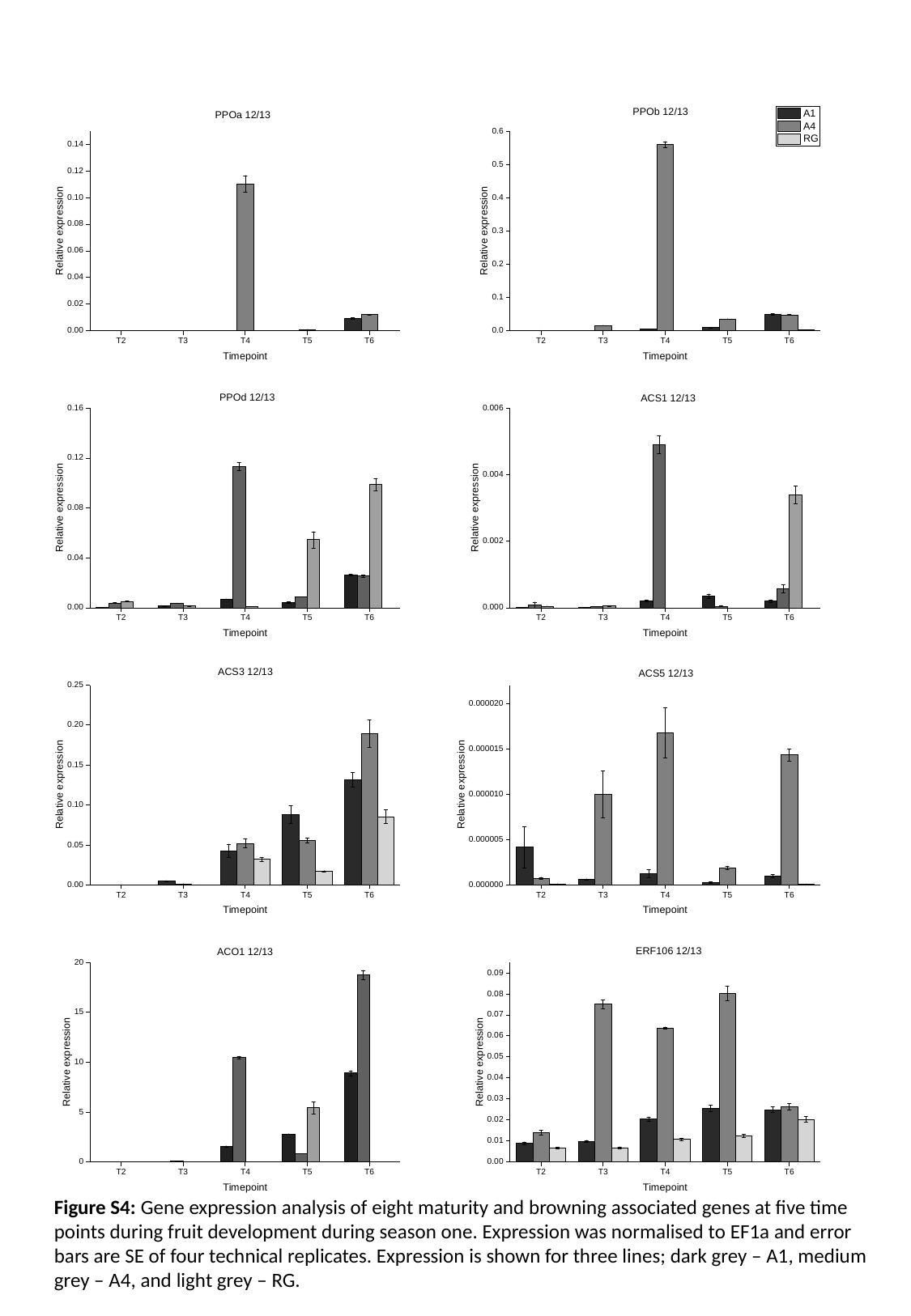

Figure S4: Gene expression analysis of eight maturity and browning associated genes at five time points during fruit development during season one. Expression was normalised to EF1a and error bars are SE of four technical replicates. Expression is shown for three lines; dark grey – A1, medium grey – A4, and light grey – RG.

## Slide 6
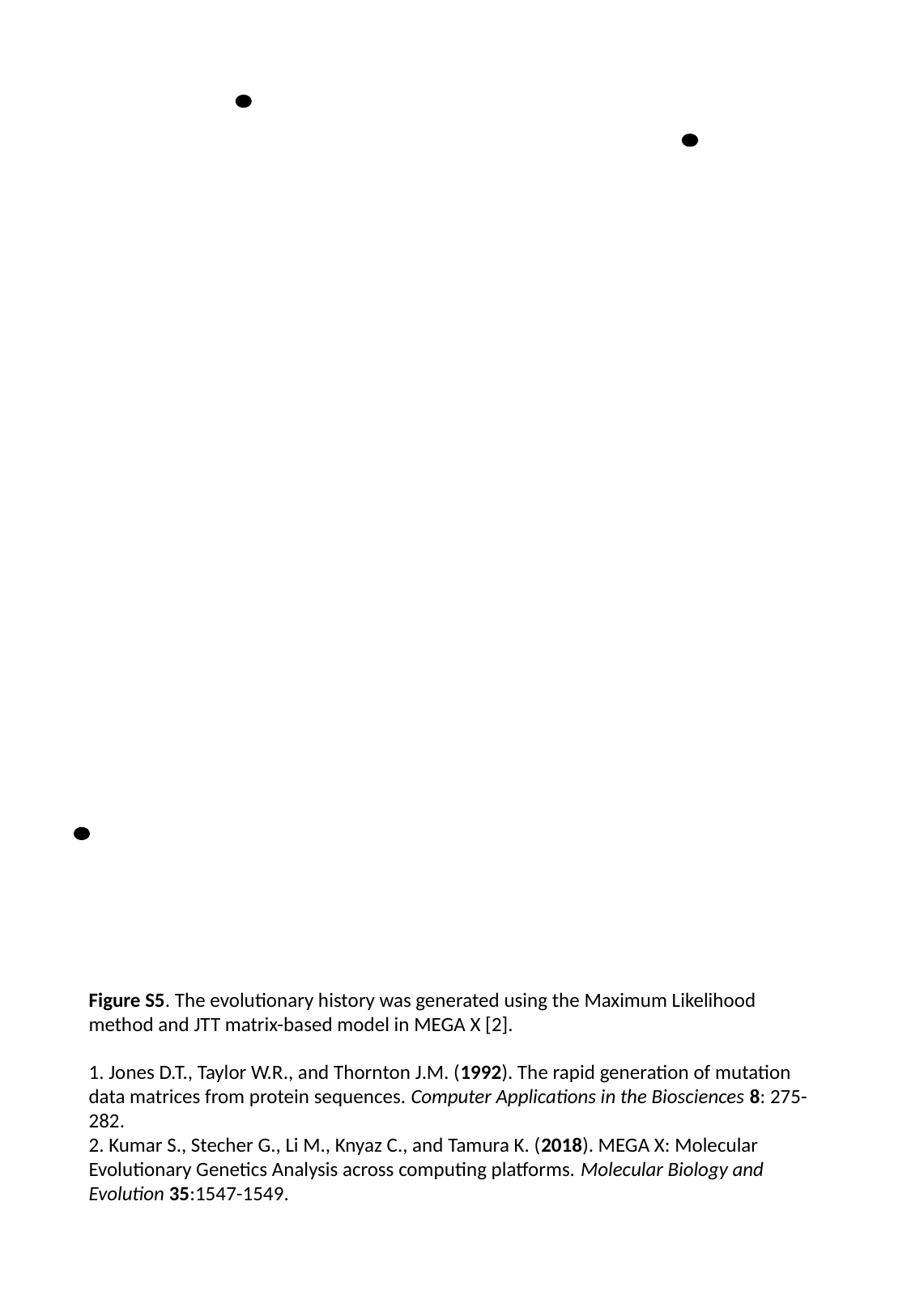

Figure S5. The evolutionary history was generated using the Maximum Likelihood method and JTT matrix-based model in MEGA X [2].
1. Jones D.T., Taylor W.R., and Thornton J.M. (1992). The rapid generation of mutation data matrices from protein sequences. Computer Applications in the Biosciences 8: 275-282.
2. Kumar S., Stecher G., Li M., Knyaz C., and Tamura K. (2018). MEGA X: Molecular Evolutionary Genetics Analysis across computing platforms. Molecular Biology and Evolution 35:1547-1549.
